# CHARACTERIZATION OF NOVEL ACCELEROMETER-MEASURED PHYSICAL ACTIVITY METRICS BY COGNITIVE STATUS IN OLDER ADULTS

**DOI:** 10.1093/geroni/igae098.3476

**Published:** 2024-12-31

**Authors:** Yurun Cai, Beth Snitz, Ann Cohen

**Affiliations:** University of Pittsburgh, Pittsburgh, Pennsylvania, United States; University of Pittsburgh, Pittsburgh, Pennsylvania, United States; University of Pittsburgh, Pittsburgh, Pennsylvania, United States

## Abstract

Although levels of physical activity (PA) are associated with risk of mild cognitive impairment (MCI) and Alzheimer’s disease (AD), most studies have used self-report PA and/or focused solely on intensities (e.g., MVPA) which may lead to recall bias or measurement error. We aim to characterize novel PA metrics among adults aged ☐50y with various cognitive status in the Connectomics in Brain Aging and Dementia study. A total of 166 (mean age 65.3☐8.9y, 65.9% women) participants with neuropsychological and valid accelerometer data in 2016-2021 were included in the analysis. PA metrics included total activity counts (TAC), logged TAC (LTAC), activity fragmentation, active minutes/day, number of active bouts, and PA complexity calculated using multiscale entropy. Participants were classified into normal control (NC)(n=84), impaired without complaints (IWOC) or subjective cognitive complaints (SCC)(n=36), and MCI/AD(n=44). Analysis of variance analysis found that IWOC/SCC had fewer number of active bouts than NC (85.34 vs. 91.83, p=0.043), lower PA complexity than NC (52.57% vs. 64.33%, p=0.001) and MCI/AD (52.57% vs. 60.68%, p=0.045). Participants with MCI/AD had lower LTAC than NC (14.38 vs. 14.50, p=0.042) and higher activity fragmentation than the other two groups (25.89% vs. 23.70%, p=0.035). Logistic regression models showed that each 1% higher in PA complexity was associated with 4% lower odds of IWOC/SCC compared to NC (OR=0.96, 95%CI:0.93-0.99, p=0.004), adjusted for sociodemographics. Our study highlights the importance of assessing novel PA metrics particularly PA complexity which may be more sensitive than conventional metrics to identify adults at early stages of pathological progression to dementia.

